# Improving redox sensitivity of roGFP1 by incorporation of selenocysteine at position 147

**DOI:** 10.1186/s13104-018-3929-x

**Published:** 2018-11-22

**Authors:** Katherine R. Stanford, Joanne M. Ajmo, Parmvir K. Bahia, Stephen H. Hadley, Thomas E. Taylor-Clark

**Affiliations:** Department of Molecular Pharmacology & Physiology, Morsani College of Medicine, University of South Florida, 12901 Bruce B Downs Blvd, Tampa, FL 33612 USA

**Keywords:** Green fluorescent protein, Reporter, Redox-sensing, Selenocysteine, Reactive oxygen species

## Abstract

**Objective:**

Redox-sensitive green fluorescent protein (roGFP) is a genetically-encoded redox-sensitive protein used to detect cellular oxidative stress associated with reactive oxygen species production. Here we replaced the cysteine at position 147 of roGFP1 (variant of roGFP) with selenocysteine in order to increase redox sensitivity of the redox reporter.

**Results:**

Expression of roGFP1 selenoprotein (roGFP1-Se147) in HEK293 cells required the presence of a selenocysteine insertion sequence and was augmented by co-expression with SBP2. roGFP1-Se147 demonstrated a similar excitation and emission spectra to roGFP1. Although expression of roGFP1-Se147 was limited, it was sufficient enough to perform live cell imaging to evaluate sensitivity to oxidation and reduction. roGFP1-Se147 exhibited a 100-fold increase in sensitivity to oxidation with H_2_O_2_ in comparison to roGFP1 as well as a 20-fold decrease in the EC_50_ of H_2_O_2_. Furthermore, roGFP1-Se147, unlike roGFP1, was able to detect oxidation caused by the mitochondrial electron transport complex III inhibitor antimycin A. Unfortunately roGFP-Se147 exhibited a diminished dynamic range and photoinstability.

**Electronic supplementary material:**

The online version of this article (10.1186/s13104-018-3929-x) contains supplementary material, which is available to authorized users.

## Introduction

Investigations into reactive oxygen species (ROS) signaling has been hindered by a lack of available methods to accurately measure small and localized ROS fluctuations. Unfortunately, redox-sensitive dyes often lack the ability to localize in sub-cellular compartments and are generally incompatible with in vivo use. Genetically-encoded reporter proteins such as redox-sensitive green fluorescent protein (roGFP) have been used in vivo and targeted to many organelles, but they lack the sensitivity of dyes [1–5]. Although roGFP2 is sensitive to hypoxia-mediated ROS production, its responses are slow [1, 6], suggesting significant limitations for the detection of local, small-scale, and transient fluctuations involved in physiologic ROS signaling.

roGFP1 is a commonly used variant of roGFP that is largely unaffected by pH and produces a strong fluorescent signal [7]. roGFP1 contains two redox-sensitive cysteines at amino acids 147 and 204 which serve as a biological switch. Oxidation and reduction of these cysteines shifts fluoresce between two excitation maxima (405 and 470 nm) [1, 8]. As such, redox state is expressed as a ratio of these excitation maxima (405/470). Upon exposure to an oxidative environment (either ROS or oxidized glutathione), the redox-sensitive cysteines are oxidized, resulting in intramolecular disulfide bond formation, increased fluorescence at 405 nm and decreased fluorescence at 470 nm (increased 405/470 ratio) [8]. Reduced glutathione reduces the disulfide bond, restoring fluorophore structure and fluorescent properties to the pre-oxidized state.

Despite these ratiometric and reversible properties, roGFP’s lack of sensitivity renders it unsuitable for detecting ROS fluctuations involved in physiologic signaling [1]. One factor contributing to its relative insensitivity is the protonation of its redox-sensitive cysteines under physiologic cellular conditions: only deprotonated cysteines are able to react with ROS [9]. To overcome this barrier, several attempts have been made to decrease the pKa (from ~ 8.2) of the roGFP1 cysteines by mutating surrounding amino acids [10]. Unfortunately, the resulting proteins also lacked the sensitivity to detect small ROS fluctuations. An alternative approach to increase the sensitivity of roGFP1 is to replace the relevant cysteines with selenocysteine, a more reactive nucleophile with a lower pKa (5.5 vs 8.2) [11]. Selenocysteine is capable of forming bridges upon oxidation with either selenocysteine or cysteine [12].

Selenocysteine incorporation into proteins is complex and inefficient compared to insertion of canonical amino acids. Selenocysteine incorporation occurs at UGA codons, which typically serve to stop protein translation [13]. Thus, incorporation requires additional elements including a selenocysteine insertion sequence (SECIS) in the 3′ untranslated region of the mRNA and a selenocysteine specific elongation factor [14–17]. Here we attempted to increase the sensitivity of roGFP1 by mutating the functional cysteine (at position 147) to selenocysteine (roGFP1-Se147). Though roGFP1-Se147 demonstrated increased ROS sensitivity, it exhibited a poor dynamic range and photoinstability.

## Main text

### Methods and results

All roGFP constructs were derived from roGFP1-N1 [7] (Additional file 1: Methods). To maximize selenoprotein expression, we used two systems for expression of the roGFP1 selenoprotein (roGFP1-Se147): pLuc01 and pSel. Both selenoprotein expression vectors (selenovectors) contained a 3′ SECIS element. To increase selenocysteine incorporation, roGFP1-Se147 was co-expressed with selenocysteine binding protein 2 (SBP2) by either co-transfection of a second plasmid (pLuc01 system) or co-expression on the same vector (pSel system). The pLuc01 system involved co-transfection of two plasmids: the LucC258U/wtP plasmid containing a 3′ PHGPx SECIS element [18], and the hSBP2/V5-HIS plasmid, contained human SBP2. The pSel system utilized a dual expression in the pSelExpress1 plasmid which contains a modified Toxoplasma gondii SECIS element and the C terminal functional domain of rat SBP2 [19].

**Fig. 1:**
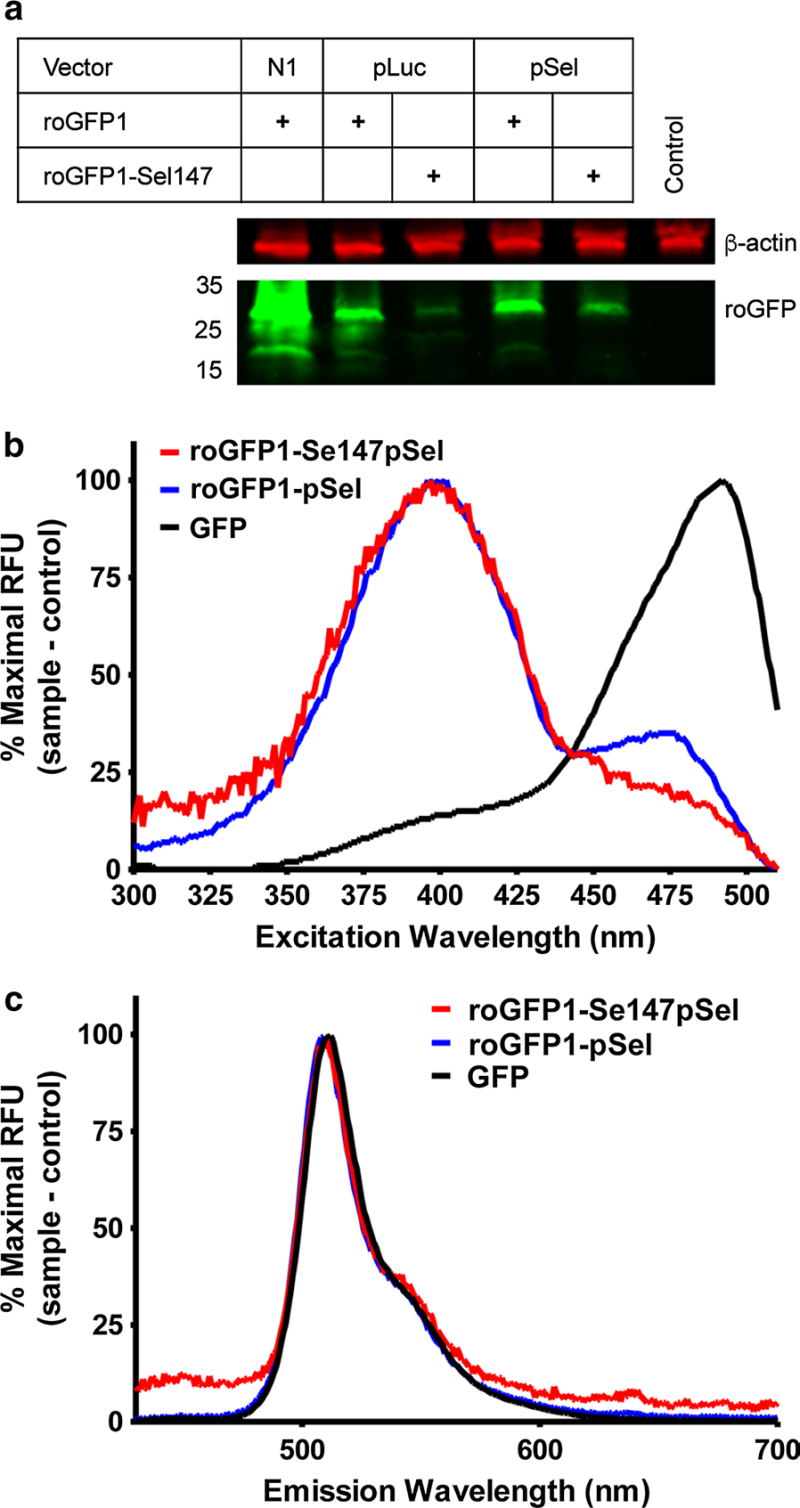
Expression and spectra of roGFP (green) constructs. Constructs expressed in HEK293T. **a** Western blot of lysates from HEK293 cells following expression of roGFP1-N1, roGFP1-pLuc, roGFP1-Se147pLuc01, roGFP1-pSel, roGFP1-Se147pSel and a non-transfected control. β-actin (red) used as a loading control. **b** Excitation spectra, measured at 530 nm. **c** Emission spectra, excited by 405 nm

HEK293T cells were transfected with roGFP1-N1 (control vector), roGFP1-pLuc01, roGFP1-Se147pLuc01, roGFP1-pSel, or roGFP1-Se147pSel (Fig. 1a). roGFP1 was highly expressed in the control vector (roGFP1-N1) as assessed by western blot. roGFP1 expression was reduced with use of both the selenovectors (roGFP1-pSel, and roGFP1-pLuc01). Expression of roGFP1-Se147 was limited but detectable for both selenovectors, although expression was consistently higher with the roGFP1-Se147pSel vector. Previous studies have demonstrated protein truncation at selenocysteine insertion sites [14, 18, 19]. However, no evidence of protein truncation was observed for the selenoproteins.

Using transfected HEK293T cells suspended in PBS, we evaluated the excitation (detected at 530 nm, Fig. 1b) and emission spectra (excited at 405 nm, Fig. 1c) of GFP, roGFP1-pSel and roGFP1-Se147pSel. To account for the differences in expression efficiency (GFP > roGFP1-pSel > roGFP1-Se147pSel), spectral data was normalized to maximal peak for each fluorescent protein. Redox-insensitive GFP exhibited a large excitation peak at ~ 480 nm and roGFP1-pSel exhibited two excitation peaks with the largest peak at ~ 405 nm and a smaller peak at ~ 475 nm, consistent with previous studies [1]. The excitation spectrum for roGFP1-Se147pSel was similar to roGFP1-pSel with its maximum at ~ 405 nm. All proteins exhibited similar emission spectra with a single maximum at ~ 530 nm.

**Fig. 2:**
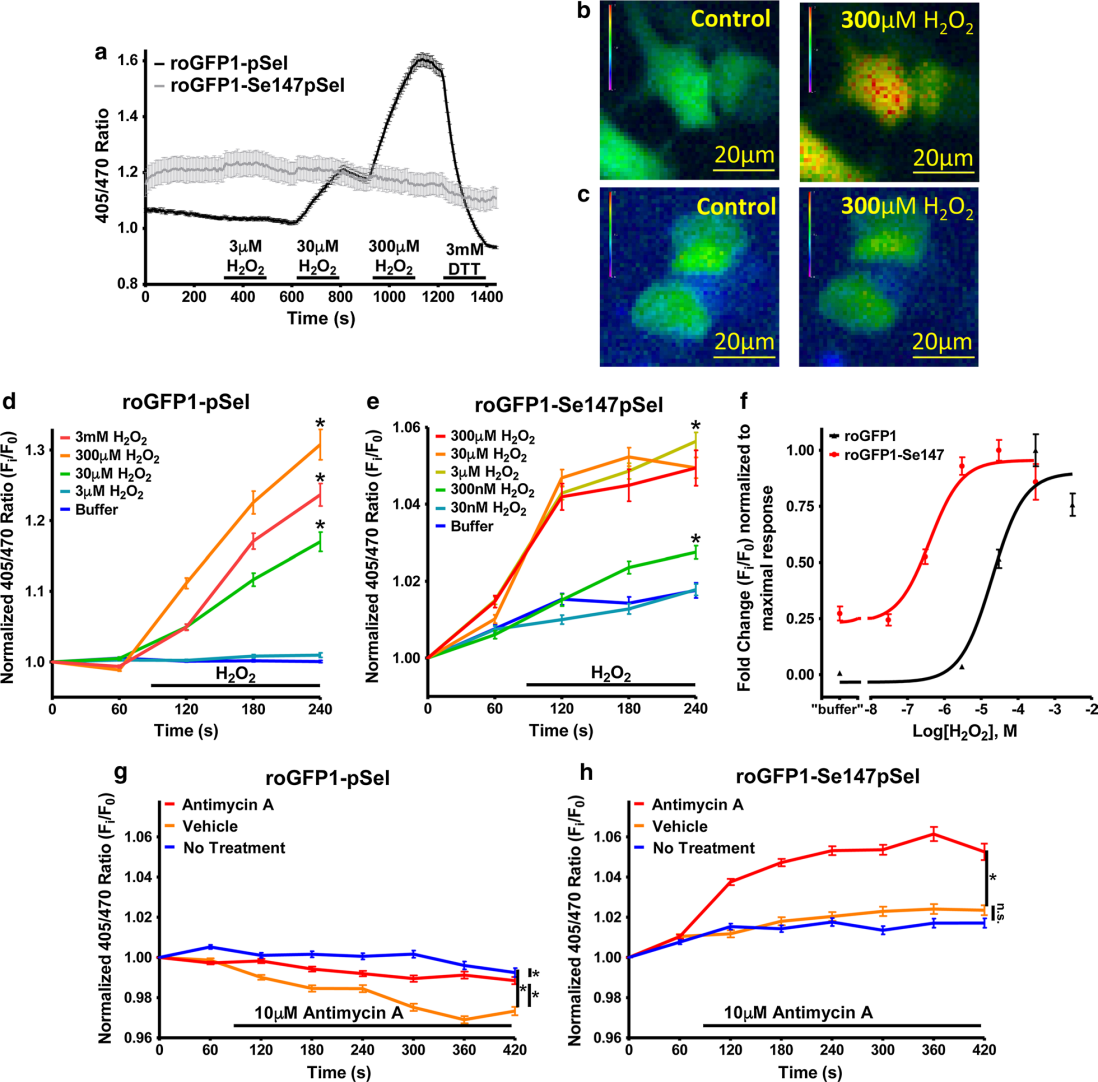
Response of roGFP1 and roGFP1-Se147 to oxidation and reduction. **a** Mean ± SEM of the 405/470 ratio of HEK293 cells transfected with roGFP1-Se147pSel (grey, n = 52) and roGFP1-pSel (black, n = 220) treated with 3, 30, and 300 µM H_2_O_2_ followed by 3 mM DTT. **b** and **c** Representative pseudocolor image of 405/470 ratio of HEK293 cells transfected with roGFP1-pSel (**b**) and roGFP1-Se147pSel (**c**) in control conditions and after treatment with 300 µM H_2_O_2_. **d** normalized mean ± SEM 405/470 response of HEK293 expressing roGFP1-pSel to 3 µM to 3 mM H_2_O_2_ (n = 62 to 263) with reduced recording frequency. *Denotes significant difference at 240 s in normalized ratio between different treatment groups (one way ANOVA, p < 0.01). **e** Normalized mean ± SEM 405/470 response of HEK293 expressing roGFP1-Se147pSel to 30 nM to 300 µM H_2_O_2_ (n = 103 to 346) with reduced recording frequency. *Denotes significant difference at 240 s in normalized ratio between different treatment groups (one way ANOVA, p < 0.01). **f** Concentration-normalized response relationship of H_2_O_2_ treatment in roGFP1-pSel and roGFP1-Se124pSel expressing HEK293 cells fitted using a non-linear regression. EC_50_ for roGFP1-pSel and roGFP1-Se147pSel was 2.0 × 10^−5^ (R^2^ of 0.94) and 9.8 × 10^−7^ (R^2^ of 0.97), respectively. **g** and **h** normalized mean ± SEM 405/470 response of HEK293 expressing roGFP1 constructs to 10 μM antimycin A (red lines), 0.1% ethanol vehicle (green lines) and buffer (blue lines). **g** roGFP1-pSel (n = 221–363). **h** roGFP1-Se147pSel (n = 237–346). *Denotes significant difference in normalized ratio between different treatment groups (repeated measures ANOVA, p < 0.01). *n.s.* denotes no significant difference between groups (p > 0.05)

Though expression of roGFP1-Se147pSel was limited, sufficient fluorescent signals were observed in individual HEK293 to evaluate the responsiveness of roGFP1-Se147pSel to oxidation and reduction. Individual construct-expressing cells were assessed (as previously [20]) for their 405/470 ratio during exposure to 3–300 µM H_2_O_2_. Omission of sodium selenite from the culture prevented roGFP1-Se147pSel expression (data not shown). roGFP1-pSel was insensitive to oxidation with 3 µM H_2_O_2_, but oxidation with 30–300 µM H_2_O_2_ evoked substantial increases in the 405/470 ratio, which was reversed by the reducing agent dithiothreitol (DTT) (3 mM) (Fig. 2a, b). The selenoprotein, roGFP1-Se147pSel, exhibited minor responses to oxidation with 3–300 µM hydrogen peroxide as well as to reduction with 3 mM DTT, indicating reduced fluorophore dynamic range (Fig. 2a, c). In comparison to roGFP1-pSel, roGFP1-Se147pSel exhibited an elevated baseline 405/470 ratio, which increased by ~ 5% under resting conditions until leveling off after 2 min, suggesting photoinstability of the selenoprotein.

In order to determine the redox-sensitivity of roGFP1-Se147pSel while minimizing any excitation-associated photoinstability, we reduced the exposure time and frequency of the sequential excitation. As the baseline for roGFP1-Se147pSel was greater than roGFP1-pSel, we normalized results (fold change baseline) to facilitate comparison between the constructs. As before, HEK293 cells transfected with roGFP1-pSel exhibited a robust response to ≥ 30 µM H_2_O_2_ (p < 0.05), but again failed to respond to 3 µM H_2_O_2_ (p > 0.05) (Fig. 2d). Whereas cells transfected with roGFP1-Se147pSel exhibited an increased normalized 405/470 ratio in response to ≥ 300 nM H_2_O_2_ (p < 0.05) (Fig. 2e). Thus the threshold for H_2_O_2_ detection for roGFP1-Se147pSel was approximately 100-fold lower than that of roGFP1-pSel. Curve fitting of the dose–response relationships showed that roGFP1-Se147pSel was approximately 20 times more sensitive to oxidation with H_2_O_2_ compared to roGFP1-pSel (EC50 of 9.8 × 10^−7^ M and 2.0 × 10^−5^ M, respectively) (Fig. 2f). Consistent with our previous data, roGFP1-Se147pSel demonstrated a greatly diminished dynamic range compared to roGFP1-pSel.

We next evaluated the sensitivity of the selenoprotein to endogenous ROS evoked by the mitochondrial complex III inhibitor antimycin A in HEK293 cells [21, 22]. Antimycin A (10 µM) failed to increase the 405/470 ratio of HEK293 transfected with roGFP1-pSel (p > 0.05, compared to untreated cells) (Fig. 2g). Whereas antimycin A increased the 405/470 ratio of HEK293 transfected with roGFP1-Se147pSel (p < 0.05), compared to the 0.1% ethanol vehicle or untreated control) (Fig. 2h). Unexpectedly, the 0.1% ethanol vehicle decreased the roGFP1-pSel 405/470 ratio (p < 0.01) but this did not occur with roGFP1-Se147pSel (p > 0.05). Overall, the data suggest that roGFP1-Se147pSel is sufficiently sensitive to detect endogenous oxidative stress produced downstream of mitochondrial dysfunction.

**Fig. 3:**
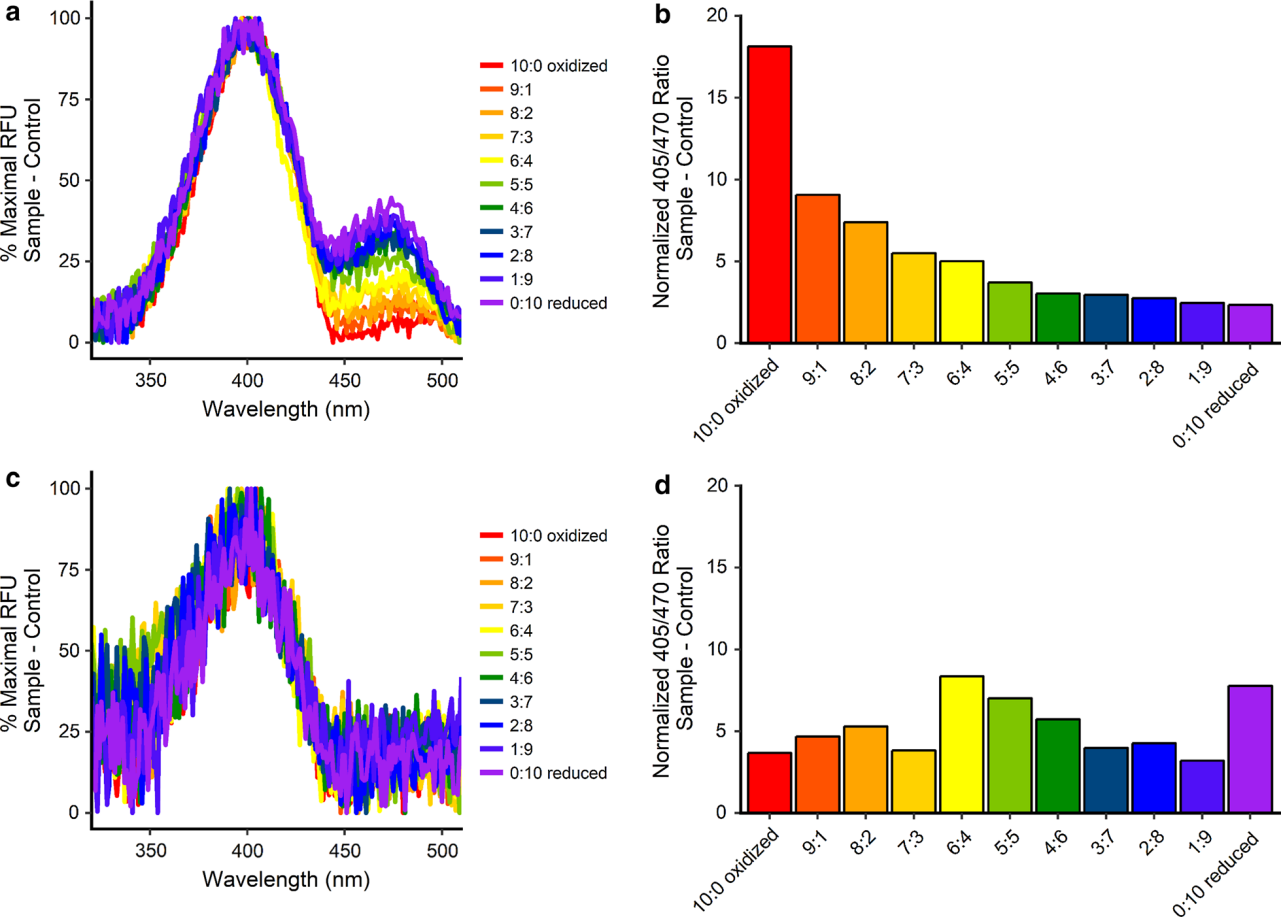
Redox titration of roGFP1 and roGFP1-Se147. Constructs expressed in HEK293T. Following purification roGFP constructs were incubated with 10 mM lipoate buffer with increasing ratios oxidized:reduced lipoate. **a** Normalized excitation spectra for roGFP1 in lipoate buffers. **b** Calculated 405/470 ratio of roGFP1 in lipoate buffers. **c** Normalized excitation spectra for roGFP1-Se147 in lipoate buffers. **d** Calculated 405/470 ratio of roGFP1-Se149 in lipoate buffers

Finally, cytosolic proteins were first collected from saponin-treated HEK293T cells transfected with either roGFP1-pSel or roGFP1-Se147pSel, then purified and concentrated using spin columns (10 kDa MW cutoff). We performed a redox titration using a 10 mM lipoate buffer with increasing ratios oxidized:reduced lipoate (from 0:10 to 10:0 in increments of 1 mM) and fluorescent spectra were obtained, yielding the 405/470 excitation ratios. As expected the spectra for roGFP1-pSel was redox sensitive (Fig. 3a, b), with a calculated redox potential of − 289.9 mV, similar to the − 288 mV calculated by other labs [8]. Purified roGFP1-Se147pSel was barely detected above background (Fig. 3c). Furthermore, the spectra indicated no consistent changes in 405/470 ratio throughout the titration (Fig. 3d), thus the redox potential of roGFP1-Se147 was not able to be calculated.

### Discussion

The mutation of cysteine to selenocysteine (UGA) at position 147 in both selenovectors yielded a full-length protein (~ 27 kDa), indicating successful incorporation of the selenocysteine. We found that the pSelExpress1 vector (containing both the selenoprotein and SBP2) produced relatively more selenoprotein, consistent with its reported increased efficiency [19]. Spectral analysis revealed similar excitation and emission spectra for both roGFP1 and roGFP1-Se147, indicating that the 405/470 ratio was appropriate for determining redox-sensitivity of roGFP1-Se147. Despite the low selenoprotein expression, sufficient roGFP1-Se147 was expressed in some HEK293 cells to perform fluorescent live cell imaging. Consistent with previous reports cells transfected with roGFP1 responded robustly to substantial oxidation caused by concentrations of ≥ 30 µM H_2_O_2_, but failed to respond to either 3 µM H_2_O_2_ or 10 µM antimycin A [1]. Cells transfected with roGFP1-Se147 demonstrated a 100-fold lower H_2_O_2_ detection threshold than those transfected with roGFP1. Furthermore, cells transfected with roGFP1-Se147 demonstrated sufficient sensitivity to detect mitochondrial ROS evoked by > 30 s treatment with the mitochondrial complex III inhibitor antimycin A. Our previous studies have shown that antimycin A causes mitochondrial ROS production and mitochondrial depolarization within 30 s [23]. Thus our data indicates increased sensitivity of the selenoprotein redox sensor to oxidation with both exogenous and endogenous ROS. Consistent with its structural similarities to GFP, roGFP1-Se147 was expressed within the cytosolic compartment.

## Limitations

Unfortunately, roGFP1-Se147 exhibited a decreased dynamic range and photoinstability. By decreasing the excitation exposure to roGFP1-Se147, we were able to resolve stimuli-induced responses. However, these characteristics limit the usefulness of this roGFP1-Se147 as an effective reporter. Both the diminished dynamic range and photoinstability can likely be attributed to unforeseen structural consequences resulting from the replacement of cysteine with selenocysteine. Other studies have shown that roGFP1 exhibits some photoswitching with prolonged exposure to 405 nm light [24]. This results in a shift toward the 470 nm excitation maximum independent of disulfide bond formation. It is possible that selenocysteine altered the protein structure in such a way that it worsened the existing photoinstability of roGFP1.

Most selenoproteins have a single selenocysteine residue [25]. Selenocysteine incorporation requires multiple elements including SECIS, a selenocysteine specific elongation factor and selenium. As such, the incorporation efficiency of selenocysteine is reduced compared to that of canonical amino acids [26–28]. Here, we found that expression levels of roGFP1-Se147 were greatly reduced but were sufficient in individual cells. Furthermore, the functionality (dynamic range, photostability) was disrupted. Previous studies have successfully inserted selenocysteine (in the place of cysteine) into proteins normally lacking selenocysteine including luciferase, rat growth hormone receptor, thyroid hormone receptor, glutaredoxin and plant phospholipid hydroperoxide glutathione peroxidase [26, 29–31]. Thus, the decreased functionality of roGFP1-Se147 is likely a specific effect on roGFP1, rather than a general property of non-native selenocysteine incorporation.

Despite the functional changes in 405/470 ratio of roGFP1-Se147 in HEK293 cells upon exposure to H_2_O_2_ and antimycin A, we were unable to demonstrate redox-induced fluorescence changes in purified roGFP1-Se147. While the decreased expression of roGFP1-Se147 hindered the spectral analysis, we believe this is not solely responsible for the lack of measurable redox sensitivity. Such a state suggests that either the selenocysteine or cysteine within the redox-sensing moiety of the roGFP have become oxidized sufficiently that it is no longer sensitive to reducing agents. We have previously shown that roGFP1 loses the ability to report redox status following cysteine covalent modification by *n*-methyl-maleimide [20]. Despite selenocysteines being more sensitive to oxidation than cysteines, cysteines are more sensitive to ‘over-oxidation’, i.e. into sulfinic and sulfonic states, than selenocysteines [32]. Indeed, the presence of a selenocysteine may increase the ‘over-oxidation’ of cysteines [33]. It is possible that the roGFP1-Se147 became irreversible oxidized during the extraction/purification protocol. Without a redox titration of roGFP1-Se147, we cannot prove that this particular roGFP construct is directly modified by redox environment. As such, we have no mechanistic evidence that the increased sensitivity of changes in 405/470 ratio of roGFP1-Se147 in HEK293 cells upon exposure to low levels of H_2_O_2_ is due to a shift in the redox potential of the redox-sensing moiety.

In conclusion, we present a selenocysteine-containing mutant of roGFP1 that displays an apparent increased sensitivity to ROS but also has decreased dynamic range and photoinstability. The development of probes with increased sensitivity is a pre-requisite for the understanding of the multiple roles of ROS and oxidative stress in physiological and pathophysiological processes.

## Additional file

**Additional file 1.** Supplemental methods. Further details of the methods used in this study.
