# Supplementary material for: Improving redox sensitivity of roGFP1 by incorporation of selenocysteine at position 147
Source: BMC Res Notes. 2018 Nov 22;11:827. doi: 10.1186/s13104-018-3929-x (PMC6249920; doi:10.1186/s13104-018-3929-x)
Supplement: Supplementary file 1 — Additional file 1. Supplemental methods. Further details of the methods used in this study. [file 13104_2018_3929_MOESM1_ESM.docx]

**Methods**

*Creation roGFP1 selenoprotein*

All roGFP constructs were derived from roGFP1-N1 (generous gift from Dr. SJ Remington, University of Oregon). To maximize selenoprotein expression, we used two systems for expression of the roGFP1 selenoprotein (roGFP1-Se147): pLuc01 and pSel. Both selenoprotein expression vectors (selenovectors) contained a 3’ SECIS element. To increase selenocysteine incorporation, roGFP1-Se147 was co-expressed with selenocysteine binding protein 2 (SBP2) by either co-transfection of a second plasmid (pLuc01 system) or co-expression on the same vector (pSel system). The pLuc01 system involved co-transfection of two plasmids: the LucC258U/wtP plasmid containing a 3’ PHGPx SECIS element (generous gift from Dr. P Copeland, Rutgers University), and the hSBP2/V5-HIS plasmid (generous gift from Dr. P Copeland, Rutgers), contained human SBP2. roGFP1 was subcloned from roGFP1-N1 into LucC258U/wtP (generous gift from Dr. P Copeland, Rutgers University) plasmid using HindIII and PacI (inserted using primer) restriction sites (roGFP1-pLuc01). Custom designed primers with a single point mutation were then used to mutate roGFP1 nucleic acid 441 from C to A, creating a UGA codon for selenocysteine insertion (roGFP1-Se147pLuc01). The pSel system utilized a dual expression in the pSelExpress1 plasmid (generous gift from Dr. V Gladyshev, Brigham and Women's Hospital, Harvard Medical School) which contains a modified Toxoplasma gondii SECIS element and the C terminal functional domain of rat SBP2. Both roGFP1 and roGFP1-Se147 were PCR amplified from the pLuc01 expression system and inserted into pSelExpress1 using XbaI and HindIII restriction sites (roGFP1-pSel and roGFP1-Se147pSel). The success of all insertions and mutations were confirmed by sequencing.

*Cell Culture*

HEK293 cells were used for functional analysis. HEK239T cells were used for all other studies to maximize protein expression. Cells were lifted using Accutase and seeded onto poly-D lysine/laminin coverslips (live cell imaging), six well plates (western) or culture flasks (spectral analysis) and cultured in Dubecco’s modified Eagle’s medium containing 4.5g/L glucose, L-glutamine, and sodium pyruvate (Corning, Manassas, VA, REF: 10-013-CV). The media was supplemented with 0.5% penicillin/streptomycin and 10% fetal bovine serum. Cells were transfected at 80% confluency cells using lipofectamine 2000 (Thermo Fisher Scientific, Waltham, MA). Cells transfected with roGFP1-Se147 constructs were supplemented with 10nM sodium selenite at the time of transfection. Cells were allowed to incubate at 37^o^C with 5% CO2 and used within 36 hours.

*Western Blot*

HEK293T Cells were transfected with roGFP1-pLuc01, roGFP1-Se147pLuc01, roGFP1-pSel, roGFP1-Se147pSel, and roGFP1-N1 (vector control). Transfected cells were lysed using lysis buffer (50mM TRIS, 150mM NaCl, 2% TritonX-100, and 0.05% SDS) and protein collected via centrifugation. Total protein was calculated using a bicinchoninic acid assay (Thermo Scientific, Rockford, IL), and samples were diluted to 1ug/ul in Licor 4X protein sample loading buffer. Protein (10µg) was loaded onto a Mini-Protean TGX 4-20% precast gel (Bio-Rad, Hercules, CA). Following electrophoresis, proteins were transferred onto a nitrocellulose membrane using a Trans-Blot Turbo transfer pack (Bio-Rad, Hercules, CA) on a Trans-Blot Turbo (Bio-Rad, Hercules, CA). Membranes were blocked in Odyssey blocking buffer (LI-COR, Lincoln, NE) for 60 minutes then left in 1:500 chicken anti GFP (Aves cat# GFP-1020) and 1:500 mouse anti β–actin (Novus, Minneapolis, MN, Cat# NB600-501) diluted in Odyssey blocking buffer + 0.15% Tween-20 overnight at 4ᵒC. Gels were washed 6x10 minutes with PBS + 0.1% Tween-20 and incubated in 1:20,000 donkey anti mouse 680 (LI-COR, Lincoln, NE, Cat# 926-68022) and 1:20,000 donkey anti chicken 800 (LI-COR, Lincoln, NE, Cat# 926-32218) diluted in in Odyssey blocking buffer + 0.15% tween +0.02% SDS. After six washes with PBS + 0.1% Tween-20, membranes were imaged using a Licor Odyssey (LI-COR, Lincoln, NE).

*Spectral analysis in HEK293T*

HEK293T cells were transfected with pEGFP-N1 (Clonetech, Mountain View, CA), roGFP1-pSel, or roGFP1-Se147pSel. To obtain a cell suspension for analysis, cells were lifted with Accutase, spun down then resuspended in 600µl PBS (250,000 cells for GFP control, and 750,000 roGFP1-pSel and roGFP1-Se147pSel transfected cells). 100µl of each cell suspension was loaded into a well on an optical 96 well plate. Scans were performed using a Synergy Mx Microplate Reader (BioTek, Winooski, VT). Excitation scans between 350-520nm were detected at 530nm, emission scans were detected between 430 and 700nm following excitation at 405nm. For analysis, background fluorescence (non-transfected cells) was subtracted from the sample fluorescence. Data was then normalized to the maximum peak.

*Functional Analysis in HEK293*

HEK293 cells on poly-D-lysine and laminin coverslips were transfected with roGFP constructs, then placed in a chamber and perfused with 10mM HEPES buffer (154mM NaCl, 1mM KCl, 0.5M MgCl_2_, 2.5mM CaCl_2_, 5.6mM D-glucose) at 33-34^ᵒ^C and evaluated for changes in cellular redox state. Drugs were diluted in HEPES (DTT, 3mM; H_2_O_2_, 30nM to 3mM; Antimycin A, 10µM). Cells were monitored using sequential excitation at 405 and 470nm every 6 or 60 seconds with emissions recorded at 510nm using microscopy (CoolSnapHQ2; Photometrics Surrey, BC, Canada), and analyzed using NIS elements software (Nikon, Melville, NY).

*Redox Titration of Purified roGFP Constructs*

HEK293T cells were transfected with roGFP1-pSel, and roGFP1-Se147pSel (see cell culture). After 24 hours, cells were lifted with Accutase, transferred to a conical tube then spun at 700 x g for 2 minutes. Each pellet was resuspended in chilled PBS + 100ug/ul Saponin + 0.5mg/ml Roche Complete Mini protease inhibitors + 1mM DTT and incubated on ice for 3 minutes. The use of saponin permitted extraction of only small cytosolic proteins (including roGFP constructs). DTT was included to protect the cysteines and selenocysteines of roGFP1-Se147pSel and roGFP-pSel from oxidation. Saponin-permeabilized cells were centrifuged at 700 x g for two minutes. The supernatant was removed and placed in a microcentrifuge tube and spun at 1400g for 10 minutes at 4^o^C. Again, the supernatant was removed, then placed in a pierce spin column (10kDa MW cutoff), volume adjusted to 6mL and centrifuged at 4^o^C at 4000 x g until the volume was reduced 90-95%. To exchange the buffer, remaining liquid was diluted to 2mL with 75mM HEPES + 125mM KCl + 1mM EDTA and centrifuged again at 4^o^C at 4000 x g until the volume was reduced by 90-95%. The remaining liquid was removed and volume adjusted to account for variations in cellular growth. This process served to remove the DTT and any cytosolic glutathione from the purified protein samples. For each condition, 180μl of sample was pipetted into each of 11 wells on a 96 well optical plate with 20μl of the appropriate lipoic acid buffer (10mM final) and incubated at room temperature for one hour.

An excitation scan (350-520nm excitation; 530nm emission) was performed using a Synergy Mx Microplate Reader (BioTek, Winooski, VT). For analysis, background fluorescence (non-transfected cells) was subtracted from the sample fluorescence. To calculate the 405/470 ratio, we used the average of 403-407 excitation and 468-472 to mitigate the impact of noise. For each titration increment (from 0:10 to 10:0) we calculated Y = (R_n_-R_min_)/(R_max_-R_n_), where R_n_ is 405/470 ratio at a specific oxidized:reduced buffer ratio, R_min_ is the minimum 405/470 ratio (0:10 reduced) and R_max_ is the maximal 405/470 ratio (10:0 oxidized). A plot of Y versus log([oxidized] / [reduced]) was then generated. The buffer ratio required to achieve 50% protein oxidation (A) was determined by calculating the Y intercept of the trendline. K_eq_ was calculated from the following equation: log K_eq_=log(F470_red_)/(F470_ox_)– log A. The equilibrium potential was calculated using the Nernst equation: E’= E’_0_ - (RT/nF) ln K_eq_, where E’ is the equilibrium potential, E’_0_ is the redox potential of the buffer (lipoate = -290 mV), R is the gas constant (8.313 J/mol/K), T is temperature (in K), n is the number of electrons exchanged (i.e. 2) and F is Faraday’s constant (96490 J/mol/V).

*Statistical Analysis*

Using SPSS (IBM, Armonk, New York), a repeated measures ANOVA was used to evaluate effects of hydrogen peroxide and Antimycin A treatments. To evaluate the effects of treatment for each time point a one way ANOVA was performed (SPSS). A p value less than 0.05 was taken as significant. Using Prism (GraphPad, La Jolla, CA), a non-linear regression was used to generate a concentration response-curve and calculate the EC50 of H_2_O_2_.
